# Patient‐derived scaffolds as a model of colorectal cancer

**DOI:** 10.1002/cam4.3668

**Published:** 2020-12-23

**Authors:** Gabrielle T. Parkinson, Simona Salerno, Parmida Ranji, Joakim Håkansson, Yalda Bogestål, Yvonne Wettergren, Anders Ståhlberg, Elinor Bexe Lindskog, Göran Landberg

**Affiliations:** ^1^ Sahlgrenska Center for Cancer Research Department of Laboratory Medicine Institute of Biomedicine Sahlgrenska Academy at University of Gothenburg Gothenburg Sweden; ^2^ Research Institutes of Sweden (RISE) Division Biosciences and Materials Section for Medical Device Technology Borås Sweden; ^3^ Department of Laboratory Medicine Institute of Biomedicine University of Gothenburg Gothenburg Sweden; ^4^ Department of Surgery Institute of Clinical Sciences Sahlgrenska University Hospital, University of Gothenburg Gothenburg Sweden; ^5^ Department of Clinical Genetics and Genomics Sahlgrenska University Hospital, University of Gothenburg Gothenburg Sweden; ^6^ Wallenberg Centre for Molecular and Translational Medicine University of Gothenburg Sweden

**Keywords:** cancer stem cells, colorectal cancer, decellularized matrix, infiltration, malignancy, patient‐derived scaffold, scaffold

## Abstract

**Background:**

Colorectal cancer is the second most common cause of cancer‐related death worldwide and standardized therapies often fail to treat the more aggressive and progressive types of colorectal cancer. Tumor cell heterogeneity and influence from the surrounding tumor microenvironment (TME) contribute to the complexity of the disease and large variability in clinical outcomes.

**Methods:**

To model the heterogeneous nature of colorectal cancer, we used patient‐derived scaffolds (PDS), which were obtained via decellularization of surgically resected tumor material, as a growth substrate for standardized cell lines.

**Results:**

After confirmation of native cell absence and validation of the structural and compositional integrity of the matrix, 89 colorectal PDS were repopulated with colon cancer cell line HT29. After 3 weeks of PDS culture, HT29 cells varied their gene and protein expression profiles considerably compared to 2D‐grown HT29 cells. Markers associated with proliferation were consistently decreased, while markers associated with pluripotency were increased in PDS‐grown cells compared to their 2D counterparts. When comparing the PDS‐induced changes in HT29 cells with clinically relevant tumor information from individual patients, we observed significant associations between stemness/pluripotency markers and tumor location, and between epithelial‐to‐mesenchymal transition (EMT) markers and cancer mortality. Kaplan–Meier analysis revealed that low PDS‐induced EMT correlated with worse cancer‐specific survival.

**Conclusions:**

The colorectal PDS model can be used as a simplified personalized tool that can potentially reveal important diagnostic and pathophysiological information related to the TME.

## Graphical abstract

Colorectal patient‐derived scaffolds (PDS) were used as culture substrate for colon cancer cell line HT29. Significant associations were observed between the PDS‐induced changes in gene expression in HT29 cells and important clinical information from individual patients. Therefore, the colorectal PDS model can potentially be used as a simplified personalized tool that can reveal important diagnostic and pathophysiological information related to the tumor microenvironment.

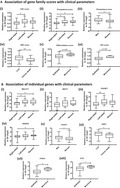

## 1 INTRODUCTION

Colorectal cancer is the third most widespread type of cancer in the world. Globally, it was estimated that 896,000 people did not survive this disease in 2017.^1^ Despite decades of intensive research, prognosis for more advanced colorectal cancer remains low.^2^ These poor patient outcomes can be partially attributed to the current lack of effective therapies to treat the huge differences that exist within individual patients, even with cancers of the same diagnosis. The individual cellular heterogeneity and tumor microenvironment (TME) in a single colorectal cancer diagnosis can potentially explain some of the varying clinical behaviors.^3^

Within the TME, the extracellular matrix (ECM) has been identified as a particularly important, but often overlooked parameter of disease progression. Indeed, colorectal cancer ECM has been shown to facilitate the development of tumorigenic vasculature, inflammatory activation, and more advanced malignancies.^4^, ^5^ 2D plastic cell culture favors the development of differentiated, actively proliferating cells, and is therefore incapable of modeling the heterogeneous nature of a natural TME that harbors cell populations of varying proliferative, stem‐like, chemo‐resistant, pluripotent, and metastatic capabilities.

The majority of artificially synthesized 3D models that currently aim to mimic the composition and support of the TME fail to replicate the complexity of TME biology and give little insight into clinical outcomes.^6^ Likewise, in vivo models of colorectal patient‐derived xenografts suffer from high failure rates and long development time, which do not align with clinical decision making.^7^ To better model colorectal cancer, we have developed a patient‐specific and TME‐inclusive growth system based on surgically resected cancer tissue from individual patients that is decellularized, and then, repopulated with standardized cancer cell lines. The removal of donor cells generated simplified model tissues, termed patient‐derived scaffolds (PDS), which allowed investigation into homogenous cell line responses to the TME and elucidation of cancer cell selected subpopulations unique to an individual tumor and patient.

The results indicated that the PDS system can be employed on colorectal tumors as easily as breast cancer tissue, which we have previously reported.^8^ By using mild detergent washes to decellularize colorectal samples, recellularization with standardized cancer cell lines was easily achieved and even non‐colorectal cell lines repopulated colorectal PDS, which demonstrated the robust nature of the PDS technique. Similar to other 3D cell culture systems, cancer cell lines grown on colorectal PDS increased the RNA expression of pluripotency markers, while proliferation markers were generally downregulated. Importantly, there was substantial variation in the cancer cell response between different PDS. These results illustrated the unique influence that an individual patient's TME has on key tumor biological pathways; and demonstrated the real‐time, clinical potential of the PDS model to monitor this in colorectal cancer.

## 2 MATERIALS AND METHODS

### 2.1 Tumor decellularization

Colorectal cancer samples were collected at the time of surgery and snap‐frozen in liquid nitrogen and stored in −80°C until use. Each tumor was dissected into 5 × 5 × 5 mm pieces. All the following wash steps were performed at 37°C in a 10L Incu‐shaker (Benchmark Scientific) with gentle shaking at 175 rpm unless otherwise stated. Tumor pieces were washed twice for 6 hours in decellularization buffer, consisting of distilled water containing 0.1% SDS (Sigma‐Aldrich), 0.02% Na‐Azide (VWR), 5 mM 2H_2_O‐Na_2_‐EDTA (Sigma‐Aldrich), and 0.4 mM phenylmethylsulfonyl fluoride (Sigma‐Aldrich). Next, tumors were rinsed in the same buffer without SDS for 15 min. Decellularized tumors were then washed for 72 h in distilled water; the total volume of water was replaced every 12 hours to remove cell debris. Tumors were then washed for 24 h in PBS (Medicago); the total volume of PBS was replaced every 8 hours. At this point, tumors were decellularized and considered as patient‐derived scaffolds (PDS). To maximize the amount of material available for experimental analysis, PDS were microdissected under sterile conditions into 1 × 1 × 1 mm pieces. Finally, PDS were sterilized by a wash in distilled water containing 0.1% peracetic acid (Sigma‐Aldrich) for 1 h at room temperature and a wash in PBS containing 1% Antibiotic‐Antimycotic (ThermoFisher Scientific) for 24 hours at 37°C. Scaffolds were stored in PBS containing 0.02% Na‐Azide and 5 mM 2H_2_O‐Na_2_‐EDTA at 4°C. Before recellularization, scaffolds were soaked in complete cell culture media for 1 hour at 37°C to remove residual storage buffer.

### 2.2 Tumor recellularization

PDS recellularization was performed with the colon cancer cell line, HT29 (ATCC HTB‐38) and breast cancer cell line MCF7 (ATCC HTB‐22). HT29 cells were cultured in McCoy's 5A modified medium, GlutaMAX supplemented with 10% fetal bovine serum and 1% penicillin/streptomycin (all ThermoFisher Scientific). MCF7 cells were cultured in Dulbecco's modified Eagle's medium (DMEM) supplemented with 10% fetal bovine serum, 1% penicillin/streptomycin, 1% nonessential amino acids, and 1% L‐glutamine (all ThermoFisher Scientific). Cells were cultured and expanded in 2D conditions to 70%–80% confluence. 5 × 10^5^ cells were added dropwise onto PDS and individually cultured in 48‐well cell culture plates (ThermoFisher Scientific) in 1 ml of cell line‐specific media supplemented with 1% Antibiotic‐Antimycotic (ThermoFisher Scientific). Seventy‐two hours after recellularization, scaffolds were transferred to a new well and visually checked for cell growth every fourth day. If cells started to proliferate onto the plastic surface beyond the scaffold, the PDS was transferred to a new well with fresh media. PDS were cultured for 21 days prior to subsequent analysis, unless otherwise stated.

### 2.3 Matrigel culture

Corning Matrigel GFR Membrane Matrix (ThermoFisher Scientific) was thawed in ice and diluted 1:2 with ice‐cold cell line‐specific medium. A 600 µL cold mixture was added to 24 wells plates and incubated at 37 C for 30 minutes to allow jellification. A total of 3x10^5^ cells were then added on top of Matrigel in 1 ml volume. Matrigel culture was carried out for 21 days and medium was replaced every second day.

### 2.4 Histology and immunohistochemistry

Tissue sections were fixed with 4% phosphate‐buffered formalin (HistoLab), dehydrated in increasing concentrations of ethanol (Solveco), infiltrated with xylene, embedded in paraffin (HistoLab), and sectioned to 4.5 µm thickness by Microm Cool‐cut (ThermoFisher Scientific). For hematoxylin and eosin (H&E) staining, sections were deparaffinized, stained with Mayer's hematoxylin (HistoLab) and 0.2% eosin (HistoLab), followed by dehydration with increasing concentrations of ethanol and mounting onto glass slides with Pertex (HistoLab). Picro‐Sirius red staining was performed using Picro‐Sirius Red Stain Kit (Abcam) according to the manufacturer's instructions. Briefly, sections were deparaffinized as before and incubated in Picro‐Sirius Red Solution for 60 minutes, quickly rinsed twice in acetic acid and twice in 100% ethanol before mounting onto glass slides with Pertex. Alcian Blue staining was performed using Alcian Blue, pH2.5 Mucin Stain Kit (Abcam) according to the manufacturer's instructions. As before, sections were deparaffinized, and then, incubated in acetic acid for 3 minutes, followed by an incubation with Alcian Blue (pH 2.5) Solution for 30 minutes. Sections were then quickly rinsed twice in distilled water, incubated in Nuclear Fast Red Solution for 5 minutes, rinsed twice again in distilled water before dehydration with increasing concentrations of ethanol and mounting onto glass slides with Pertex.

Immunohistochemistry was performed using Autostainer LINK 48 (DAKO) using the Envision FLEX+detection system (DAKO). Briefly, sections were subjected to antigen retrieval by heating samples to 97°C for 10 min in EnVision™ FLEX target retrieval solution high pH (DAKO), followed by blocking with EnVision™ FLEX Peroxidase‐Blocking Reagent (DAKO) for 10 min and incubation with primary antibody for 1 hour (primary antibody list is documented in supplementary Table S6). For signal amplification, the EnVision™ FLEX+Mouse linker (DAKO) was incubated with samples for 30 minutes, followed by EnVision™ FLEX/HRP visualization reagent (DAKO) for 30 min, and EnVision™ FLEX DAB+Chromogen (DAKO) for 5 minutes. Antibody stained slides were counterstained with EnVision™ FLEX hematoxylin (DAKO). Stained sections were dehydrated and mounted as before, and then, scanned by Leica SCN400 scanner (Leica Microsystems) at 40× resolution.

### 2.5 Cell viability assays

Both 2D‐grown and PDS‐grown HT29 cells were detached with Accutase (ThermoFisher Scientific) incubation at 37°C for 5 minutes, centrifuged at 300× *g* for 3 min and the cell pellet was resuspended. Viability was assessed using the Trypan Blue dye exclusion assay (Sigma), where cells were stained with a 1:1 mixture of 0.4% trypan blue; viable cells that excluded the dye and the unviable cells that contained the dye were counted. Viability was calculated using the formula: (Unviable cells per ml/Viable cells per ml) × 100.

Cell metabolic activity was measured over the course of 72 hours with the Alamar Blue colorimetric assay (Sigma). Following detachment, 2D‐ and PDS‐grown HT29 cells were plated at the same density in 2D culture and incubated at 37ºC. Alamar Blue solution was formulated in a ratio of 0.5 mg/10 ml in a serum‐free media. The plates were washed with PBS and 100 µl of the Alamar Blue solution was added to each well and incubated for 1 h at 37ºC before measurements were taken at 2, 4, 6, 48, and 72 hours post‐seeding. The excitation and emission fluorescence of the dye was measured at a wavelength of 540 vs 590 nm, respectively, with VICTOR Multilabel Plate Reader.

### 2.6 DNA and RNA extraction

DNeasy Blood and Tissue kit (Qiagen) was used to extract DNA from colorectal PDS following overnight air‐drying at room temperature and homogenization in 100 µl PBS in a rotor‐stator homogenizer. DNA concentration was measured using the Qubit 3 fluorometer (ThermoFisher Scientific).

For RNA extraction, 2D‐grown cells and recellularized PDS were washed twice with PBS and lysed directly in RLT buffer (Qiagen). Matrigel‐grown cells were lysed in Qiazol (Qiagen). RNA was then extracted directly or samples were placed on dry ice and stored in −80°C. Samples were then thawed on ice and homogenized using a stainless steel beads (Qiagen) in TissueLyzer II (Qiagen) for 5 minutes at 25 Hz. Samples were visually inspected for complete homogenization, if not 5 minutes additional homogenization was repeated until achieved. RNA was extracted using miRNeasy Mini Kit (Qiagen), including DNase treatment (Qiagen). RNA concentration was measured by NanoDrop (ThermoFisher Scientific) and RNA quality was randomly assessed using Bioanalyzer 2100 (Agilent Technologies).

### 2.7 Reverse transcription quantitative PCR

Complimentary DNA synthesis from RNA was performed using GrandScript cDNA synthesis kit (TATAA Biocenter). Reverse transcription was performed on a T100 Thermal Cycler (Bio‐Rad) in 20 µl reaction mixes at 22°C for 5 minutes, 42°C for 30 minutes, and 85°C for 5 minutes followed by cooling to 4°C until subsequent analysis. RNA Spike II (TATAA) was added in the reverse transcription reaction as quality control. All samples were diluted 1:3 with RNsse‐free water (ThermoFisher Scientific) before further processing. Quantitative PCR was performed on a CFX384 Touch Real‐Time PCR Detection System (Bio‐Rad) using 1x SYBR GrandMaster Mix (TATAA), 400 nM of each primer (see supplementary Table S7), and 2 µl diluted cDNA in a final reaction volume of 6 µl. The temperature profile was 95°C for 2 minutes followed by 35–50 cycles of amplification at 95°C for 5 seconds, 60°C for 20 seconds, and 70°C for 20 seconds and a melting curve analysis at 65°C to 95°C with 0.5°C per 5 s increments. All samples were validated by melting curve analysis. Cycles of quantification values were determined by the second derivative maximum method with the CFX Manager Software version 3.1 (Bio‐Rad). Cycle of quantification values larger than 35 were replaced with 35 and missing data were replaced with a value derived from the imputation of other scaffold replicate values. Gene expression was normalized using reference genes identified with the NormFinder algorithm. Then, cycle of quantification values were transformed to relative quantities through normalization to 2D controls and log2 transformed. Data preprocessing was performed using GenEx (MultiD). All experiments were conducted in accordance with the Minimum Information for Publication of Quantitative Real‐Time PCR Experiments (MIQE) guidelines.^9^

### 2.8 Western blotting

Samples were diluted in 2x Laemmli sample buffer (10% SDS, 0.5 M Tris‐HCl pH6.8, 0.5% bromophenol blue, 10% 2‐β‐mercaptoethanol) to a final concentration of 1x, boiled for 5–10 min at 95°C and briefly vortexed. After brief centrifugation to spin down any condensed vapor, samples were electrophoresed or stored at −20°C. Protein separation was performed through sodium dodecyl sulfate polyacrylamide gel electrophoresis according to standard methods ^10^ and using Bio‐Rad Mini‐Protean II equipment. Membranes were removed from the transfer apparatus and placed in blocking buffer (3%–5% (w/v) milk/1xPBST (137 mM NaCl, 2.7 mM KCl, 10 mM Na2HPO4, 1.8 mM KH2PO4; all from Sigma) for 1 hour on a shaker at room temperature. Blocking buffer was removed and primary antibodies appropriately diluted in blocking buffer (supplementary Table S6) were incubated with the membrane for an additional hour at room temperature with shaking. The membranes were rinsed five times in blocking buffer, and then, incubated for 30–45 minutes in the appropriate horseradish peroxidase conjugated secondary antibody diluted in blocking buffer. Membranes were then washed four times under rotation for 10 minutes in 1xPBST before they were developed with the enhanced chemiluminescence western blotting detection system. Films were scanned at 600 dpi and saved as TIFF files. Images were analyzed by scanning densitometry using ImageJ (NIH).

### 2.9 Experimental design and statistical analysis

All data are presented as mean ±SEM unless otherwise stated. For experiments with n numbers lower than 8, unpaired two‐tailed Student's *t*‐tests were used for statistical comparisons between two groups, and for experiments containing three or more groups, one‐way analysis of variance (ANOVA) and Tukey's post hoc test were employed. These statistical tests were performed on GraphPad Prism (v6.0). For the analysis of patient material data sets, nonparametric univariate and multivariate analysis as well as survival analysis were carried out in SPSS Statistics (v25.0, IBM). Mann–Whitney U test was used to statistically compare two groups. Kruskal–Wallis test with Dunn's post hoc test was used to statistically compare three groups. The Benjamini–Hochberg method was employed to correct multi‐testing errors for nonparametric univariate analysis. The threshold of false discovery rate was selected to be 10%. For correlations between HT29 and MCF7 response in colorectal PDS, Spearman's rank correlation was used. Cancer‐Specific Survival (CSS) was modeled with Kaplan–Meier analysis, defining the events as death by colorectal cancer and comparing the differences with the log‐rank test. Multivariate analysis of independent predictive factors of CSS was performed using Cox proportional hazard regression model. Differences were considered significant if *p* < 0.05.

## 3 RESULTS

### 3.1 Colorectal tumor decellularization is achieved through mild detergent washes, while ECM structure and integrity is maintained

**Figure 1:**
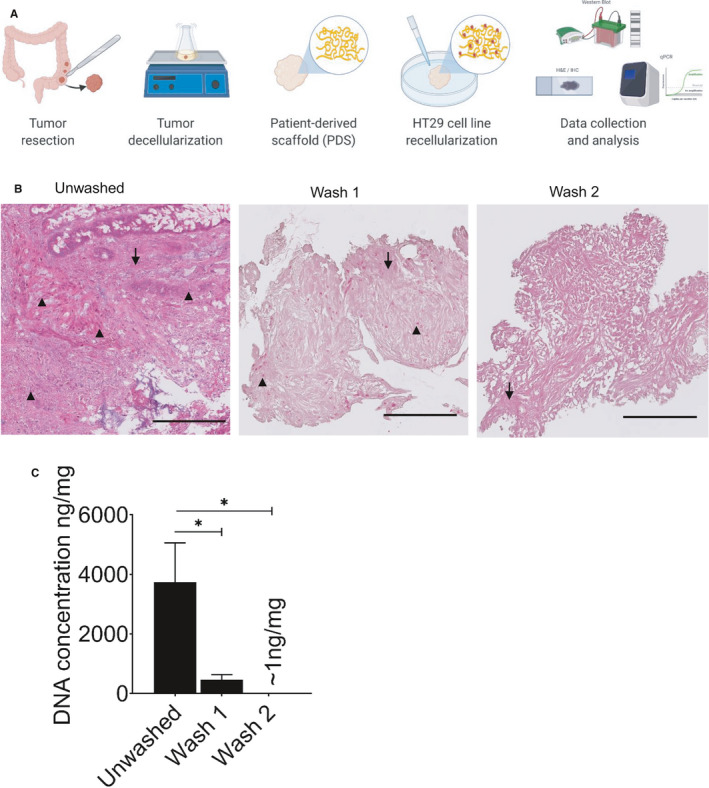
Colorectal tumor decellularization. (A) Schematic illustration of colorectal tumor decellularization workflow (created with Biorender.com). (B) H&E stained 4.5 µm sections of colorectal unwashed tumors and PDS after each cycle of decellularization. Black arrowheads point at cell nuclei and black arrows point toward intact collagen fibers. Scale bars indicate 200 µm. (C) DNA concentration measured from 5 × 5 × 5 mm unwashed (n = 2) and decellularized tumors following the first (n = 4) and second wash (n = 10). Values represent mean ±SEM, *p < 0.05 (one‐way ANOVA with Tukey post hoc test)

In the PDS model, tumor decellularization is essential prior to cancer cell line repopulation in order to exclusively monitor responses to components of the colorectal TME in the adapting cancer cell line without potential interference from patient's own cells. In the current literature, a limited number of studies have employed this technique and a variety of different decellularization methods have been developed. Here, we utilized mild detergent washes to quickly and cheaply decellularize colorectal tumors (see Figure 1A for schematic of workflow). Due to the protein denaturing properties of SDS detergent, we had to ensure that the ECM integrity and composition was adequately maintained. As illustrated in Figure 1B, two mild detergent buffer washes were sufficient to remove all donor cells and debris from 5 × 5 × 5 mm tumors, as visualized by H&E staining. Cell nuclei were observed throughout the unwashed tumor but were less frequent after wash 1 and 2 (Figure 1B). In addition to the histological evidence, a gradual decrease in the concentration of residual DNA was found after consecutive washes (Figure 1C). The obvious presence of collagen fibers as illustrated in Figure 1B also indicated a preserved ECM integrity in the cell‐free scaffolds that were generated.

**Figure 2:**
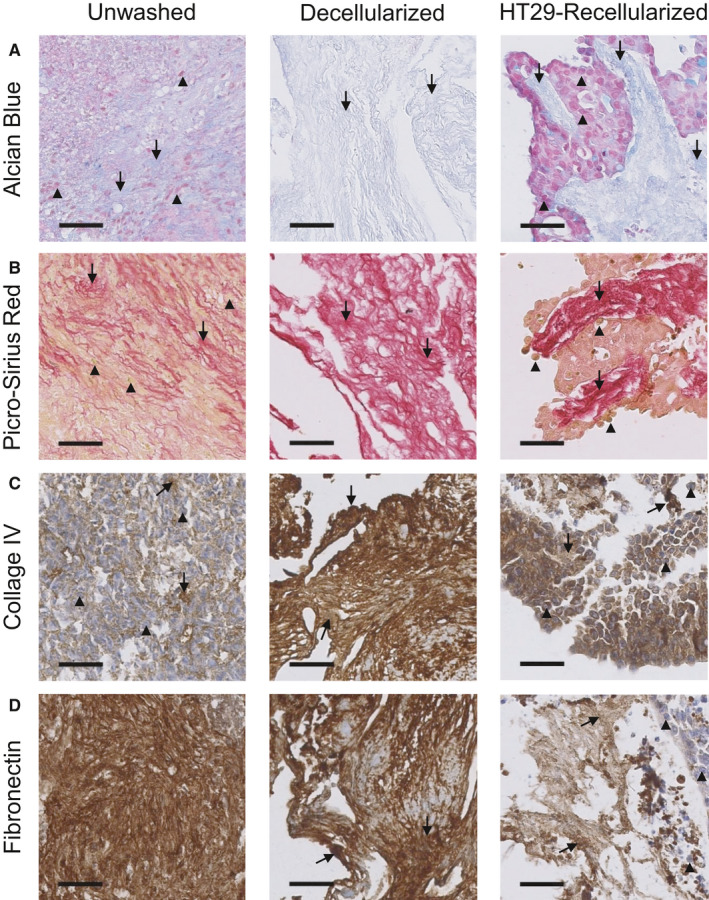
Structural and compositional characterization of decellularized and HT29‐recellularized colorectal PDS. Representative images of stained 4.5 µm sections of colorectal tumor tissue prior to washing (unwashed), after two mild detergent washes (decellularized) and after 21 days of repopulation with HT29 cell line (HT29‐recellularized). Black arrowheads point at cell nuclei and black arrows point toward intact protein structures. Scale bars indicate 200 µm. (A) Histological images of acidic polysaccharides stained with Alcian Blue. (B) Histological images of collagen I and III fibers stained with Picro‐Sirius Red. (C) Immunohistochemical images of collagen IV protein. (D) Immunohistochemical images of fibronectin protein

Given that the PDS model is reliant upon the dynamic interaction between cells and tumor tissue, it was essential to verify that important ECM proteins were preserved throughout the de‐ and recellularization process. The presence and integrity of selected ECM proteins that are commonly expressed in colorectal cancer tissues were evaluated using traditional histological staining and immunohistochemistry on preprocessed colorectal tumor tissue, de‐ and recellularized tissue. Alcian Blue was used to detected acidic polysaccharides, such as glycosaminoglycans, which are essential mediators of cancer cell biology,^11^ and Picro‐Sirius Red staining was used to detect the presence of collagen I and III fibers. The presence of glycosaminoglycans was unaffected by the PDS production process, as intense blue staining was still detectable throughout the ECM in de‐ and recellularized tissue with a distribution and organization comparable to preprocessed samples (Figure 2A). Similarly, intense pink‐red Picro‐Sirius staining of the ECM was observed throughout the PDS production process indicating that collagen fibers remained intact during decellularization and subsequent recellularization (Figure 2B). The additional staining methods further substantiated the complete decellularization of the colorectal tumor tissue following two mild detergent washes, since cell nuclei were notably absent in decellularized sections.

To more specifically detect proteins in the PDS model, immunohistochemistry of key colorectal ECM proteins was performed on preprocessed colorectal tumor tissue, de‐ and recellularized samples. Consistent with the traditional staining data, collagen IV (Figure 2C) and fibronectin (Figure 2D) proteins could be detected by immunohistochemistry throughout the PDS process.

The decellularization protocol employed here had a high success rate, with 89 usable PDS obtained from a batch of 100 tumor samples. Sterilization was also successful, as no bacterial contamination was observed in either decellularized or recellularized PDS.

### 3.2 Standardized cancer cell lines repopulate colorectal PDS after 21 days of culture

**Figure 3:**
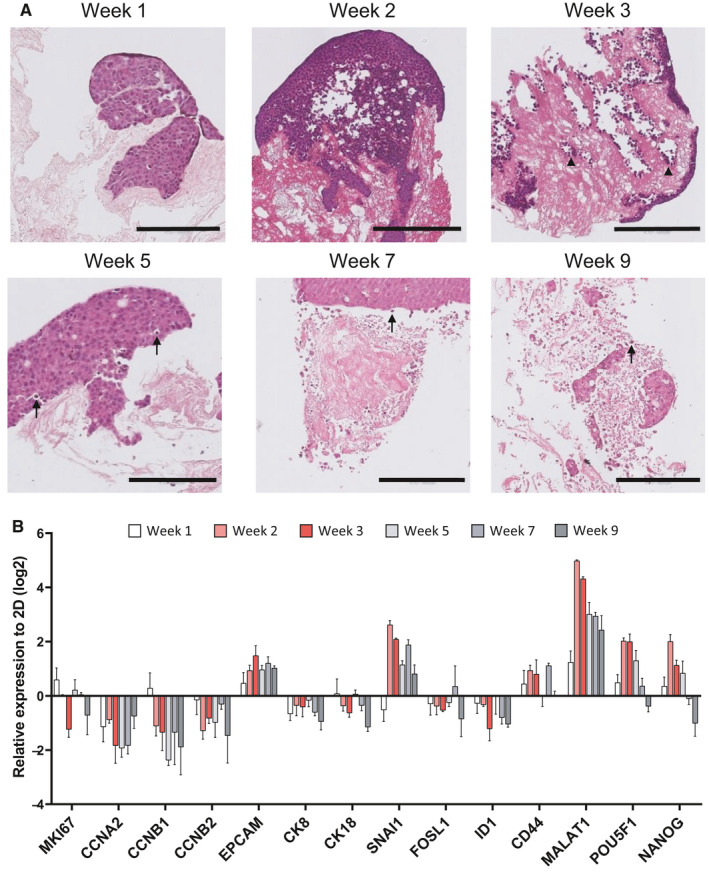
HT29 cells time‐growth on colorectal PDS. (A) Representative histological images of H&E stained 4.5 µm sections colorectal PDS after 1–9 weeks of cell culture with HT29 cells. Black arrowheads indicate endothelial cell line lining of colorectal PDS vascular structures and black arrows indicate point toward apoptotic cells. Scale bars indicate 200 µm. (B) Temporal changes in gene expression in PDS‐grown HT29 cells during 1–9 weeks of culture. Values represent mean ±SEM (n = 3)

HT29 colorectal cancer cells were chosen as an initial reference cell line as they do not display any definable features of a single consensus molecular subtype and so do not sufficiently represent a particular type of colorectal cancer.^12^ To identify optimal culture periods, HT29 cells were grown on colorectal PDS for up to 9 weeks and samples were taken for histological examination at weeks 1, 2, 3, 5, 7, and 9 (Figure 3A). After 1 week, HT29 cells were robustly attached to the colorectal PDS in irregular spheroid clusters. At weeks 2–5, HT29 cells were flattened against the colorectal PDS and formed lines of cells in former vascular spaces in a manner similar to endothelial cells and reminiscent of vascular cell morphology. (Figure 3A). During weeks 7–9, HT29 cells displayed smaller, more rounded nuclei and apoptotic bodies (Figure 3A). Matrix degradation at the cell–matrix interface started to appear at week 5.

### 3.3 Colorectal PDS induce gene and protein changes in HT29 cells

PDS‐induced changes in the RNA expression of the adapting HT29 cells were quantified over weeks 1–9. RNA expression of genes related to proliferation (*MKI67*, *CCNA2*, *CCNB1*, and *CCNB2)*, differentiation (*EPCAM*, *CK8*, and *CK18)*, epithelial‐to‐mesenchymal transition (EMT) (*SNAI1*, *FOSL1*, and *ID1)*, cancer stemness (*CD44* and *MALAT1)*, and pluripotency *(POU5F1* and *NANOG*) were quantified in 2D‐grown cells and cells grown on colorectal PDS.

Consistent with temporally distinct changes to cell line morphology, transcriptomic PDS‐induced changes of some genes were dependent on the duration of time by which cells were grown on scaffolds. Between week 1 and week 2 of HT29 PDS culture, the majority of genes displayed large changes in their expression compared to their 2D counterparts, which appeared to remain stable during weeks 2–3 (Figure 3B). During weeks 5–7, there was a second wave of transcriptomic responses and many genes exhibited a switch in direction of the PDS‐induced RNA expression compared to weeks 2–3. Furthermore, HT29 cell genetic responses to PDS‐growth altered again by week 9 compared to all other weeks.

**Figure 4:**
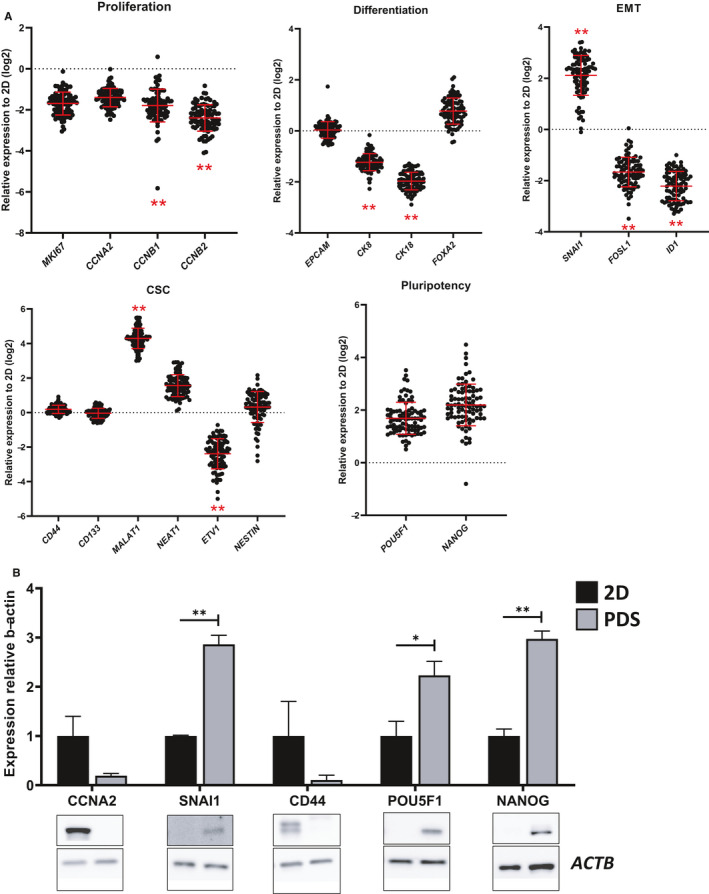
Colorectal PDS‐induced gene and protein changes in HT29 cells. (A) Gene expression fingerprint of HT29 cells (determined with qPCR) after 3 weeks growth on colorectal PDS. Data are expressed relative to 2D expression levels. Red bars indicate mean ±SD (n = 89). Dots indicate individual patients. *p < 0.05; **p < 0.01 (2D vs. PDS, Mann–Whitney U test). (B) Protein expression (determined with western blot) in PDS‐grown HT29 cells relative to 2D grown cells. Values represent mean ±SEM (SNAI1, NANOG: n = 3; CCNA2, CD44, POU5F1: n = 4), *p < 0.05; **p < 0.01 (unpaired Student's *t*‐test)

Given the unstable morphological and gene expression changes that occurred during weeks 1–2 and 5–9 of HT29 colorectal PDS cell culture, week 3 was considered as the optimal period for PDS cell growth and all of the following PDS experiments were cultured for 3 weeks. With the PDS model sufficiently validated, experiments were scaled up and HT29 cells were seeded onto all 89 PDS with varying clinical features (supplementary Table S1). As illustrated in Figure 4A, genes associated with proliferation (*MKI67*, *CCNA2*, *CCNB1*, and *CCNB2*) were downregulated in HT29 cells grown on colorectal PDS compared to their 2D counterparts. HT29 cells further displayed an increased expression of pluripotency markers (*POU5F1* and *NANOG*) once grown on colorectal PDS, compared to 2D cells, although statistical significance was not reached. However, genes associated with cell differentiation (*EPCAM*, *CK8*, *CK18*, and *FOXA2*), EMT (*SNAI1*, *FOSL1*, and *ID1*), and cancer stem cells (*CD44*, *CD133*, *ETV1*, *MALAT1*, *NEAT1*, and *NESTIN*) displayed a more varied response compared to 2D cells. For example, cytokeratins (markers of epithelial tissue) CK8 and CK18 displayed a consistent decrease in their RNA expression, while FOXA2 (a transcription factor regulating the differentiation of intestinal goblet cells ^13^) displayed a trend of increase in RNA expression in PDS‐grown HT29 cells. Moreover, the RNA expression of EMT marker SNAI1 significantly increased compared to 2D‐grown HT29 cells, whereas FOSL1 and ID1 exhibited a significant decrease in their RNA expression. Finally, cancer stem cell markers CD44, CD133, and NESTIN displayed a similar averaged RNA expression to 2D grown cells. Other cancer stem cell markers such as MALAT1 and NEAT1 were upregulated in PDS‐grown cells, while expression of ETV1 was downregulated in PDS‐grown cells compared to 2D‐grown cells.

To confirm that the observed PDS‐induced gene expression profile was not only due to 3D culturing conditions, but also the gene expression of PDS‐cultured cells was compared with that of Matrigel. The heatmap in supplementary Figure S1 summarizes the unsupervised grouping analysis of gene expression data of HT29 cells cultured in 2D, Matrigel, and PDS. This analysis showed a distinct separation of both 3D models from 2D cultures, indicating a pronounced “3D effect” in the gene expression profile. Such “3D effect” consisted in an overall decrease in the expression of proliferation genes and an overall increase in the expression of pluripotency and stemness genes. In addition, within the 3D models, Matrigel samples grouped separately from PDS samples, due to a differential expression of gene markers CCNB1, CCNB2, SNAI1, ID1, and NANOG. Statistical analysis of gene expression differences between these different models is summarized in supplementary Table S2.

To test whether these PDS‐induced transcriptomic effects were influencing changes at the proteomic level, the protein expression of key genes in PDS‐grown HT29 cells was investigated and compared to their 2D‐grown counterparts. Western blotting of HT29 cells grown on a subset of the 89 colorectal PDS for 3 weeks mirrored the transcriptomic PDS‐induced responses (Figure 4B). The proliferation marker CCNA2 showed nearly 80% reduction in its protein expression compared to 2D‐grown cells. Consistent with the changes to RNA expression levels, EMT marker SNAI1 increased its protein expression by up to threefold compared to 2D‐grown controls. Pluripotency genes, POU5F1 and NANOG, displayed approximately 2–3 fold increases in their protein expression in HT29 cells grown on colorectal PDS compared to 2D, whereas other cancer stem cell markers, such as CD44 decrease by approximately 90%.

Although, gene and protein expression information could be extracted from HT29 cells grown on colorectal PDS, it was important to ensure that cells remained viable after PDS growth. Given that 3D cell culture generally presents many obstacles to the observation of cell behavior, it was often necessary to detach HT29 cells from colorectal PDS before the following experiments could be performed. First, trypan blue viability was used to give a quick indication of whether cell detachment from colorectal PDS or plastic resulted in comparable levels of cell death. Cell detachment resulted in a small but significant difference between the proportion of live and dead cells from 2D‐ to PDS‐grown HT29 cells, with 2D‐grown cells being more viable than PDS‐grown cells after detachment (supplementary Figure S2A). Detached cells were then subjected to Alamar blue assay to investigate cell viability via measurements of metabolic activity. HT29 cells grown on colorectal PDS displayed similar values to 2D‐grown cells until approximately 48 h after plating. After 72 h PDS‐grown cells displayed a sixfold increase in metabolic activity (supplementary Figure S2B).

MKI67 staining was next employed on paraffin‐embedded PDS samples to determine the extent of active proliferation of HT29 cells between weeks 1–3 (supplementary Figure S2C).^14^ The majority of HT29 cells expressed *MKI67* illustrating a substantial fraction of actively proliferating cells despite a clear downregulation of proliferation‐associated genes compared to 2D‐grown cells, both at mRNA and protein level, during PDS adaptation as earlier showed.

### 3.4 Colorectal PDS‐induced gene expression can be linked to clinical information

To evaluate if the PDS‐induced gene expression changes in HT29 cells could offer novel molecular insight into the factors that contribute to colorectal cancer progression and clinical outcomes, qPCR analysis of cells grown on 89 colorectal PDS was statistically tested against the clinical parameters available for each PDS. The colorectal samples were selected from consecutive patients who underwent surgery in 2013 and had at least 5 years of clinical follow‐up time (supplementary Table S1). All stages and tumor locations were included and patients with preoperative chemotherapy or radiotherapy were excluded.

**Figure 5:**
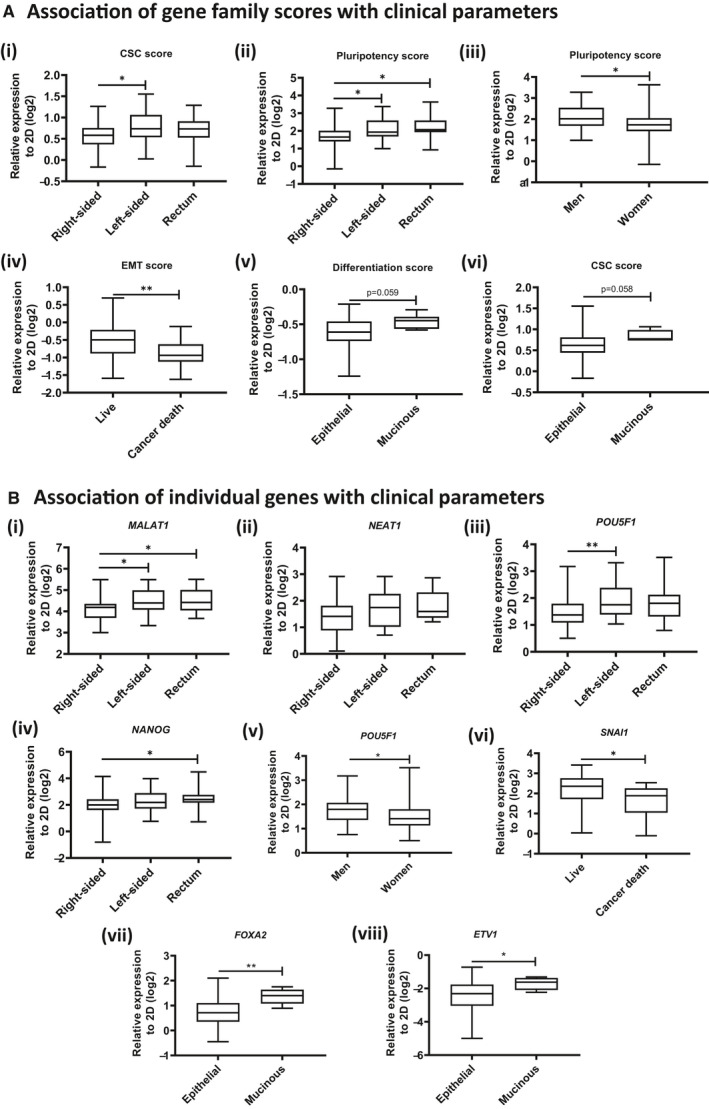
Nonparametric statistical analysis of associations of PDS‐induced HT29 gene expression response with clinical parameters. (A) Associations of gene family scores of PDS‐grown HT29 cells with (i–ii) tumor location, (iii) gender, (iv) death by cancer, and (v–vi) mucinous identity. *p < 0.05; **p < 0.01 ((i–ii) Kruskal–Wallis test with Dunn's post hoc test; (iii–vi) Mann–Whitney U test) (B) Associations of individual gene responses in PDS‐grown HT29 cells with (i–iv) tumor location, (v) gender, (iv) death by cancer, and (vii–viii) mucinous identity. *p < 0.05; **p < 0.01 ((i–iv) Kruskal–Wallis test with Dunn's post hoc test; (v–viii) Mann–Whitney *U* test)

To obtain an overview of how different aspects of PDS‐induced cell behavior correlate to clinical parameters, we averaged the RNA expression values of gene markers belonging to the same family. Hence, a single RNA expression score for each gene family was obtained for each patient. Nonparametric, univariate analysis was performed to identify potential significant associations between clinical parameters and the extent of PDS‐induced transcriptomic change of gene family scores in HT29 cells. The resulting *P*‐values are summarized in Table 1. Interestingly, several significant associations were observed between tumor location and PDS‐induced cancer stem cell (CSC) score (Figure 5A(i)), between tumor location and PDS‐induced pluripotency score (Figure 5A(ii)), between gender and PDS‐induced pluripotency score (Figure 5A(iii)), and between cancer‐specific death and PDS‐induced EMT score (Figure 5A(iv)). Furthermore, PDS derived from mucinous tumors had a tendency to induce a higher differentiation score (*p* = 0.059, Figure 5A(v)) and a higher CSC score (*p* = 0.058, Figure 5A(vi)) in HT29 cells compared to PDS derived from tumors of epithelial origin.

**Table 1:** Nonparametric statistical analysis of associations of tumor clinical parameters with PDS‐induced response of cancer‐related gene families in HT29 cells

	Tumor Location	Gender	Different.	T‐stage	Mucinous	Metastasis	N‐stage	Death by cancer
Proliferation	0.957	0.4	0.177	0.313	0.087	0.842	0.17	0.797
Differentiation	0.803	0.503	0.573	0.474	0.059	0.199	0.598	0.572
EMT	0.741	0.423	0.466	0.184	0.974	0.18	0.696	0.007
CSC	0.024	0.203	0.78	0.471	0.058	0.883	0.763	0.22
Pluripotency	0.005	0.017	0.921	0.303	0.502	0.625	0.867	0.78

The table reports *P*‐values resulting from Mann–Whitney *U* test (gender, differentiation, mucinous, metastasis, and death by cancer) or Kruskal–Wallis test (tumor location, T‐stage, and N‐stage). Proliferation family included gene *markers* *MKI67*, *CCNA2*, *CCNB1*, and *CCNB2*. Differentiation family included gene markers *EPCAM*, *CK8*, *CK18*, and *FOXA2*. EMT family included gene markers *SNAI1*, *FOSL1*, and *ID1*. CSC family included gene markers *CD44*, *CD133*, *ETV1*, *MALAT1*, *NEAT1*, and *NESTIN*. Pluripotency family included gene markers *POU5F1* and *NANOG*.

Within the CSC and pluripotency gene families, *NEAT1*, *MALAT1*, *NESTIN*, and *POU5F1* were upregulated to a significantly greater extent in PDS derived from rectal and left‐sided colon tumors compared to PDS derived from right‐sided colon tumors (Figure 5B(i‐iv)). Of these genes, MALAT1 and POU5F1 remained significantly associated to tumor location after multi‐testing correction (supplementary Table S3). RNA expression of *POU5F1* was also significantly higher in PDS resected from male patients compared to female patients (Figure 5B(v)). Within the EMT family, RNA expression of *SNAI1* was significantly higher in patients with a favorable prognosis compared to patients who died of colorectal cancer (Figure 5B(vi)). Finally, RNA expression of differentiation marker *FOXA2* and CSC marker *ETV1* was higher in PDS derived from mucinous tumors compared to PDS derived from epithelial tumors. FOXA2 remained significantly associated to the mucinous nature of the tumor after multi‐testing correction (supplementary Table S3).

**Figure 6:**
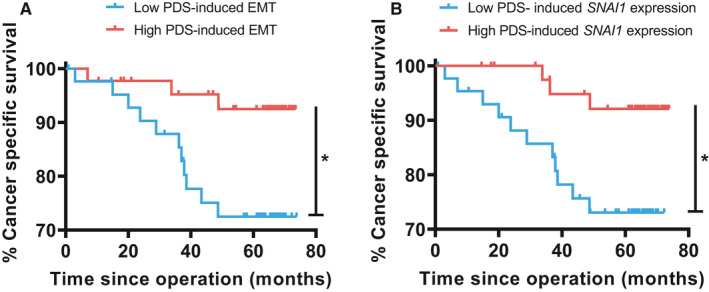
Univariate Kaplan–Meier modeling of cancer‐specific survival. (A) Correlation of PDS‐induced EMT score with CSS. *p < 0.05. (B) Correlation of PDS‐induced expression of EMT‐regulator SNAI1 with CSS. *p < 0.05

Given its association with cancer death, the EMT gene family scores were grouped into a “high” and “low” PDS‐induced response using the median value as cutoff. Kaplan–Meier analysis of CSS within these two groups revealed that low PDS‐induced EMT was significantly more likely to result in death by colorectal cancer (Figure 6A). In particular, within the EMT family, low PDS‐induced expression of EMT‐regulator SNAI1 was significantly associated with worse CSS (Figure 6B).

Next, Cox regression analysis was performed and the strong prognostic factor tumor stage (T‐stage) was included as a covariate (Table 2). Although T‐stage was the strongest predictive factor in this model, PDS‐induced EMT expression produced independent and significant predictive information. A Cox regression analysis including more covariates is summarized in supplementary Table S4.

**Table 2:** Multivariate Cox proportional hazard regression model predicting cancer‐specific survival

Covariate	Cancer‐Specific Survival		
	Hazard Ratio	Confidence interval	*p*‐value
*T‐stage*	3.456	2.186–5.465	<0.001
*PDS‐induced EMT*	0.267	0.073–0.973	0.045

### 3.5 Colorectal PDS can be repopulated with breast cancer cell line MCF7

To challenge the robustness of the PDS method, we seeded breast cancer cell line MCF7 onto colorectal PDS. MCF7 cells were able to attach and grow for 3 weeks on colorectal PDS (as evidenced by histological images in supplementary Figure S3A). During the first week of PDS culture, MCF7 cells appeared to attach over a greater surface area compared to HT29 cells. MCF7 cells initially formed sheets with a thickness of a few cells, in contrast to the spheroid‐like growth of HT29 cells. However, by week 3, due to the nature of this breast cancer cell line,^15^ the extent of invasiveness of MCF7 cells appeared to be more moderate compared to HT29 cells. RNA expression analysis showed a varied response in the direction and magnitude of transcriptomic response between genes of the proliferation and EMT families. RNA expression of differentiation genes were, overall, slightly downregulated, while the RNA expression of CSC and pluripotency genes were generally upregulated in colorectal PDS‐grown compared to 2D‐grown cells (supplementary Figure S3B(i‐v)). The gene expression profile of PDS‐grown MCF7 cells did not appear to correlate significantly to the gene expression profile of PDS‐grown HT29, as determined by Spearman correlation, shown in supplementary Figure SB(vi). Nevertheless, there were significant links between PDS‐induced MCF7 gene expression changes and the same clinical variables which were found significant in HT29 cells. In particular, PDS derived from right‐sided tumors elicited a significant downregulation of the differentiation score (supplementary Figure S3C(i)) and a nearly significant upregulation of the CSC score (supplementary Figure SC(ii)) compared to PDS derived from left‐sided tumors. Furthermore, mucinous PDS elicited a greater downregulation of differentiation score in comparison to epithelial PDS (supplementary Figure S3C(iii)). The complete nonparametric analysis of gene marker family scores within MCF7 cells and clinical parameters is summarized in supplementary Table S5. On the individual gene level, within the differentiation family of genes, CK8 and CK18 expression was downregulated to a significantly greater extent in PDS derived from mucinous tumors. However, none of the genes comprising the differentiation and CSC families were significantly linked to tumor location (supplementary Figure S4).

## 4 DISCUSSION

### 4.1 Colorectal PDS is a viable experimental model system

The use of 3D cell culture models in colorectal cancer research is gaining popularity^16^ in replacement of conventional 2D culture models lacking physiological relevance. Tissue decellularization is one of a plethora of methods to obtain 3D scaffolds^17^ and is reliant on various methods to remove host cells from surgically resected tissue. Decellularized matrices have been obtained from many organs and tissues and used in a variety of applications,^18^, ^19^ including colorectal cancer research.^20^ In contrast to other types of 3D scaffolds, cancer cells growing in a decellularized tumor are exposed to a patient‐specific TME and potentially subjected to microenvironmental cues that can drive tumor progression, but are hard to artificially mimic or are currently unknown.

Two fundamental requirements of a tissue decellularization protocol are the complete removal of donor cells and the preservation of both ECM structure and functionality. Both requirements were met by the decellularization technique used in this study, which was suited for both fresh and frozen tumor material. Although SDS has been reported to damage extracellular matrix components in a number of different tissues,^21^ our adjusted decellularization protocol efficiently removed cellular components while preserving glycosaminoglycans, collagen, and fibronectin fibers, as evidenced by histological and immunohistological stainings. Furthermore, the observation that PDS‐induced HT29 gene expression response was highly associated with patient clinical parameters was an additional indication that the PDS model retained its bioactive properties to a significant extent after decellularization.

HT29 cells appeared to attach to the PDS during the first week of culture. In addition to proliferation on the PDS, cells tended to migrate to the bottom of cell culture plate throughout the 3 weeks long PDS culture. Therefore, to exclude 2D‐growing cells and avoid their interference with downstream analysis, it was crucial to transfer the PSD to new cell culture plates every time cell feeding occurred (one or two times per week). When 2D‐ and PDS‐grown cells were detached from their respective growth substrate, PDS‐grown cells displayed a reduced viability compared to 2D‐grown cells, as evidenced by trypan blue exclusion assay. We suggest that the reduced cell viability was not resulting from a hostile PDS environment, but rather from a more firm cell adhesion to the PDS, which in turn resulted in a greater perturbation of cell viability once the cells were detached. However, this effect was small and did not obscure the higher metabolic activity of PDS‐grown cells observed with Alamar blue assay 72 hours after detachment.

Consistent with previously published studies,^22^ HT29 cells grown in PDS showed dynamic adaptation of their morphology and gene expression to the PDS environment. In particular, cells displayed a highly proliferative phase during the first week of culture, which then transitioned into a more stable phase by week 3, where cells appeared to have acquired a migratory phenotype (Figure 3).

The decreased proliferation between weeks 2–3 (observed both visually in histological sections and at the RNA level) could be attributed to the fact that most of cell adhesion sites on the PDS surface were occupied. With no available space on the surface of the PDS, HT29 cells reduced their rate of proliferation and began to migrate and invade the matrix. Nevertheless, when PDS‐grown cells were transferred back to a less crowded 2D environment, they displayed an increased metabolic activity (possibly resulting from increased proliferation) after 72 hours compared to cells that have always been cultured in 2D.

Our data support the already existing evidence that 3D culture conditions favor the invasive behavior of colorectal cancer cells, which has been demonstrated for spheroid cultures,^23^ synthetic scaffolds ^24^ and also decellularized matrices.^20^, ^25^, ^26^ In particular, matrices derived from colon tumor tissue promote migratory cell behavior to a greater extent than normal colon tissue ^20^, ^26^ or Matrigel,^25^ corroborating the evidence that the tumor microenvironment plays a significant role in cancer invasiveness.

EMT and invasiveness are often associated with cancer cell stemness.^27^ CSC are defined as a subpopulation of cancer cells with self‐renewing and tumor‐initiating capability.^28^ CSC are believed to be responsible for therapy resistance, relapse, and metastatic spread of a tumor.^29^, ^30^ Therefore, their presence in a cell culture model is crucial in order to understand these important aspects of cancer biology and in turn more accurately determine the efficacy of cancer therapies. Although colorectal CSC are commonly studied in spheroid models,^31^ these are cell line dependent ^32^ and do not account for the complexity of the TME, which has been frequently identified as an important promoting factor in CSC enrichment.^23^, ^33^, ^34^ We have previously reported that PDS derived from breast cancer tissue promote CSC activity in different breast cancer cell lines.^8^ We have now shown that the colorectal PDS model enhances the expression of some stemness and pluripotency genes, which further supports the physiological relevance of our PDS model in in vitro studies.

The reduced proliferation and the CSC enrichment observed in the PDS model can be largely attributed to the passage from 2D to 3D culturing conditions. However, significant differences in the expression of several genes were found between Matrigel and PDS cultures, suggesting that different microenvironments offer different stimuli to the cultured cell lines.

### 4.2 Colorectal PDS mirror clinical disease features

The seminal finding in this study was that the gene expression of PDS‐grown HT29 cells was highly associated with particular clinical information.

Left‐ and right‐sided colon tumors are known to be clinically and pathologically distinct ^35^ and have different prognoses.^36^, ^37^ Interestingly, this distinction was reflected by coherent changes to the RNA expression of several CSC and pluripotency markers in HT29 cells cultured on clinically distinct colorectal PDS. It has been suggested that marked differences in TME between right‐ and left‐sided colon tumors contribute to their distinct clinical and pathological features. Right‐sided colon tumors are characterized by the presence of cancer‐associated bacterial biofilms, which are absent in nearly all left‐sided colon tumors.^38^ Although the SDS washes used in our model system likely destroyed the bacteria, it is possible that bacterial secretion products were preserved to some extent during tissue decellularization and affected the gene expression adaptation of HT29 cells to the different PDS environments. Indeed, pluripotency score was significantly reduced in right‐sided compared to and left‐sided PDS. Right‐sided colon cancer patients are more likely to be women than left‐sided colon cancer patients,^35^ hence, the more moderate increase of pluripotency score in colorectal PDS derived from female patients in this study.

Mucinous colorectal adenocarcinoma is a distinct subtype of colorectal cancer, which is histologically defined by the presence of extracellular mucins comprising ≥50% of the tumor volume.^39^ TME differences between epithelial and mucinous tumors were also most likely preserved during tissue decellularization; significant differences in the RNA expression of differentiation and CSC genes were observed between HT29 cells cultured on colorectal PDS derived from epithelial or mucinous tumors.

In addition to being able to reproduce clinical variability in vitro, the reported PDS model holds the potential to deliver precious predictive information on colorectal cancer progression. High *SNAI1* expression has been consistently associated with advanced tumor stage^40^ and worse CSS.^41^ In this study, both nonparametric analysis and Kaplan–Meier analysis showed that a low PDS‐induced EMT score and a low PDS‐induced *SNAI1* expression were associated with worse CSS. The apparent discrepancy between our results and the current literature could be partly explained by an observation done by Kroepil et al.,^42^ which reported that low stage tumors had higher SNAI1 expression than advanced tumors in the tumor center but not in the invasive front. Therefore, our PDS model might mimic the transcriptomic behavior of cells in the tumor center. Furthermore, it is worth noting that the gene expression profile of a cell line does not necessarily mirror that of the patient's own cells or even other cell lines. Indeed, Stankevicius et al ^23^ have shown that HT29 cells have a different gene expression profile compared to another cell line (DLD1) when cultured as spheroids, which indicates that the changes to gene expression in response to 3D culture environments is largely cell line dependent.

In our study, some interesting associations between clinical parameters and PDS‐induced transcriptomic responses in HT29 cells were also observed in the parallel PDS experiments using the breast cancer cell line MCF7. Although some consistent changes were observed in the gene expression profiles of PDS‐grown HT29 and MCF7 cells, the direction and magnitude of such changes in relation to 2D‐grown cells was often different between cell lines. Interestingly, the comparison between HT29‐ and MCF7‐growth in colorectal PDS highlighted the possibility that although colorectal PDS can support the growth of cell lines derived from different cancer types, the cell line that is more relevant and similar to the original cancer would probably deliver information with more robust statistical significance.

## 5 CONCLUSION

Colorectal PDS are viable substrates for the growth of standardized cell lines, and can enhance the migratory features of cultured cells. Furthermore, when used as a growth substrate for HT29 cells, colorectal PDS can induce transcriptomic and proteomic responses in these cancer cells that align with patient‐specific clinical disease information. The major benefit of the colorectal PDS model is that the influence from the TME can be reliably monitored and modeled in individual cancer patients through the use of fresh or frozen tumor material within a few weeks. The PDS model can potentially provide crucial predictive information on the effectiveness of cancer therapies and consequently assist in clinical decision making in a patient‐specific manner.

## 6 ETHICAL APPROVAL AND CONSENT TO PARTICIPATE

The regional ethical review board in Gothenburg approved the study and informed consent was obtained from all patients (ethical board number 590–15).

## CONFLICT OF INTEREST

GL and AS are board members and shareholders of Iscaff Pharma, and AS is a shareholder of TATAA Biocenter. The patient‐derived scaffold approach and data are patent pending.

## AUTHORS’ CONTRIBUTIONS

GL, AS, JH, and YB conceptualized the patient‐derived scaffold model; YW and EBL provided patient material; GTP and GL designed the study; GTP, SS, and PR performed experiments and data analysis; GL, GTP, SS, YW, and EBL participated in the interpretation of the data; GTP and SS wrote and revised the manuscript; GL, AS, JH, YB, and EBL acquired funding for the study.

## Funding information

This research was funded by Assar Gabrielssons Research Foundation; Johan Jansson Foundation for Cancer Research; Knut and Alice Wallenberg Foundation, Wallenberg Centre for Molecular and Translational Medicine, University of Gothenburg, Gothenburg, Sweden; Swedish Cancer Society (19‐0306); Swedish Research Council (2016‐01530, 2017‐01392); the Swedish state under the agreement between the Swedish government and the county councils, the ALF‐agreement (716321, 721091, 784211); Wilhelm and Martina Lundgren Foundation for Scientific Research, Cancerfonden (2019‐0317), VINNOVA (2017‐03737), and Västra Götalandsregionen (2019‐01273).

## Supporting information

Supplementary Material

## Data Availability

The data presented in this study are available from the corresponding author on reasonable request.
