# Supplementary material for: Patient‐derived scaffolds as a model of colorectal cancer
Source: Cancer Med. 2020 Dec 23;10(3):867–82. doi: 10.1002/cam4.3668 (PMC7897946; doi:10.1002/cam4.3668)
Supplement: Supplementary file 1 — Supplementary Material [file CAM4-10-867-s001.docx]

#
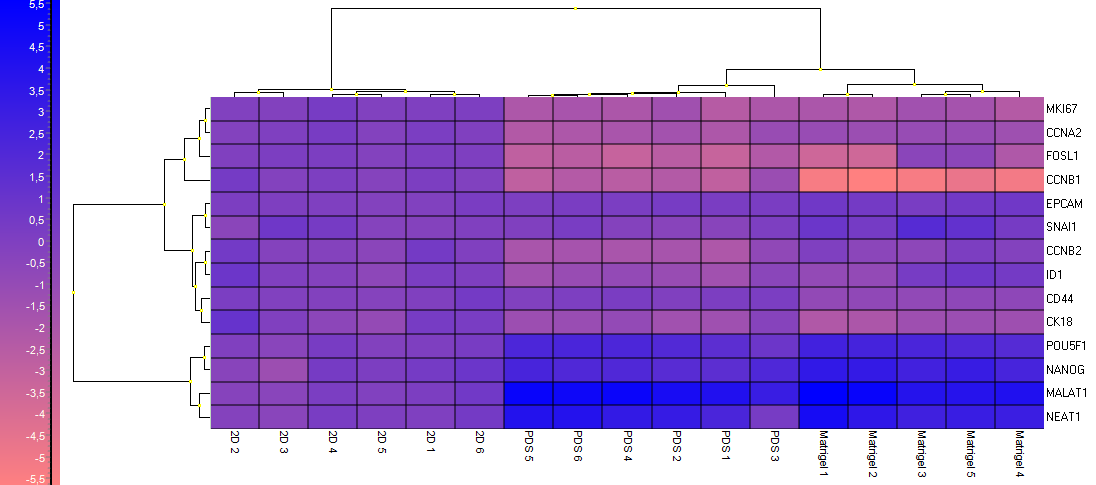
**SUPPLEMENTARY FIGURES**

**Figure S1: Gene expression differences between 2D and 3D cultures.** Unsupervised grouping analysis of gene expression data of HT29 cells cultured in 2D (n=6), Matrigel matrix (n=5) and PDS (n=6).

#
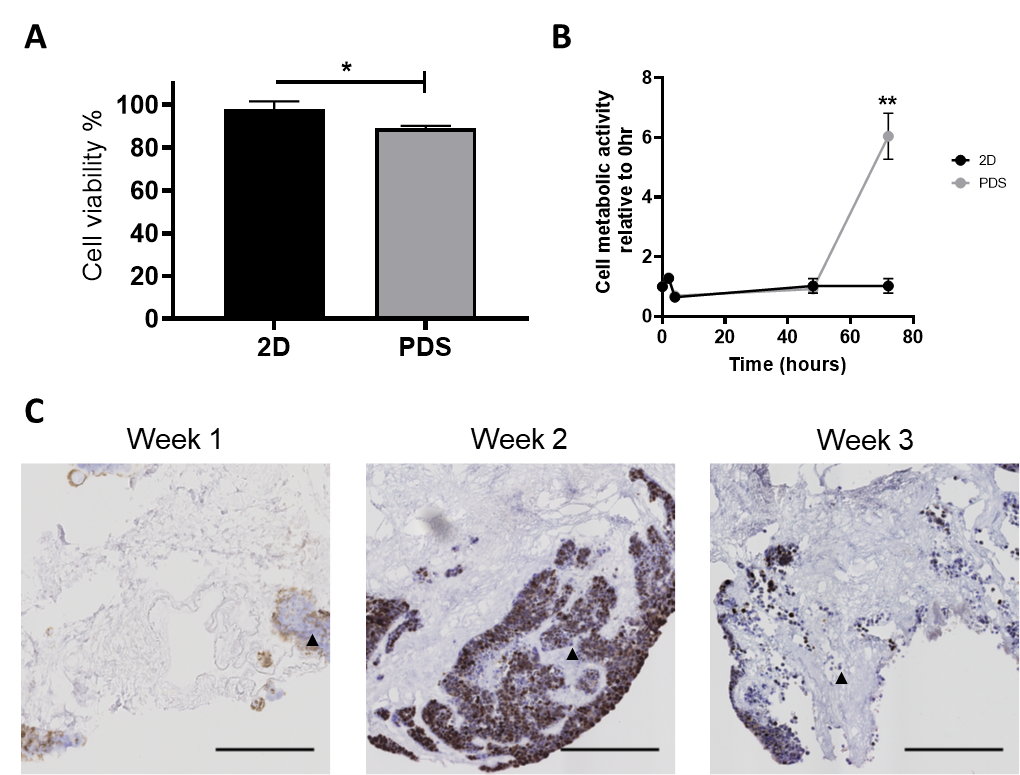


**Figure S2: Viability and proliferation of HT29 cells on colorectal PDS. (A)** Percentage of viable cells following detachment from plastic culture plate (2D) and PDS measured with trypan blue exclusion assay. Values represent mean ± SEM (n=3), *p<0.05 (unpaired Student’s t-test). **(B)** Quantification of metabolically active cells with alamar blue viability assay over the course of 72 hours. The assay was performed on cells detached from the original environment (2D or PDS) and replated in 2D. Values represent mean ± SEM (n=5), **p<0.01 (two-way ANOVA with Sidak’s post hoc test). **(C)** Immunohistological images of MKI67 protein expression in HT29 cells throughout weeks 1-3 of PDS growth. Black arrowheads indicate the presence of cell nuclei lacking MKI67 protein. Scale bars indicate 200 µm.


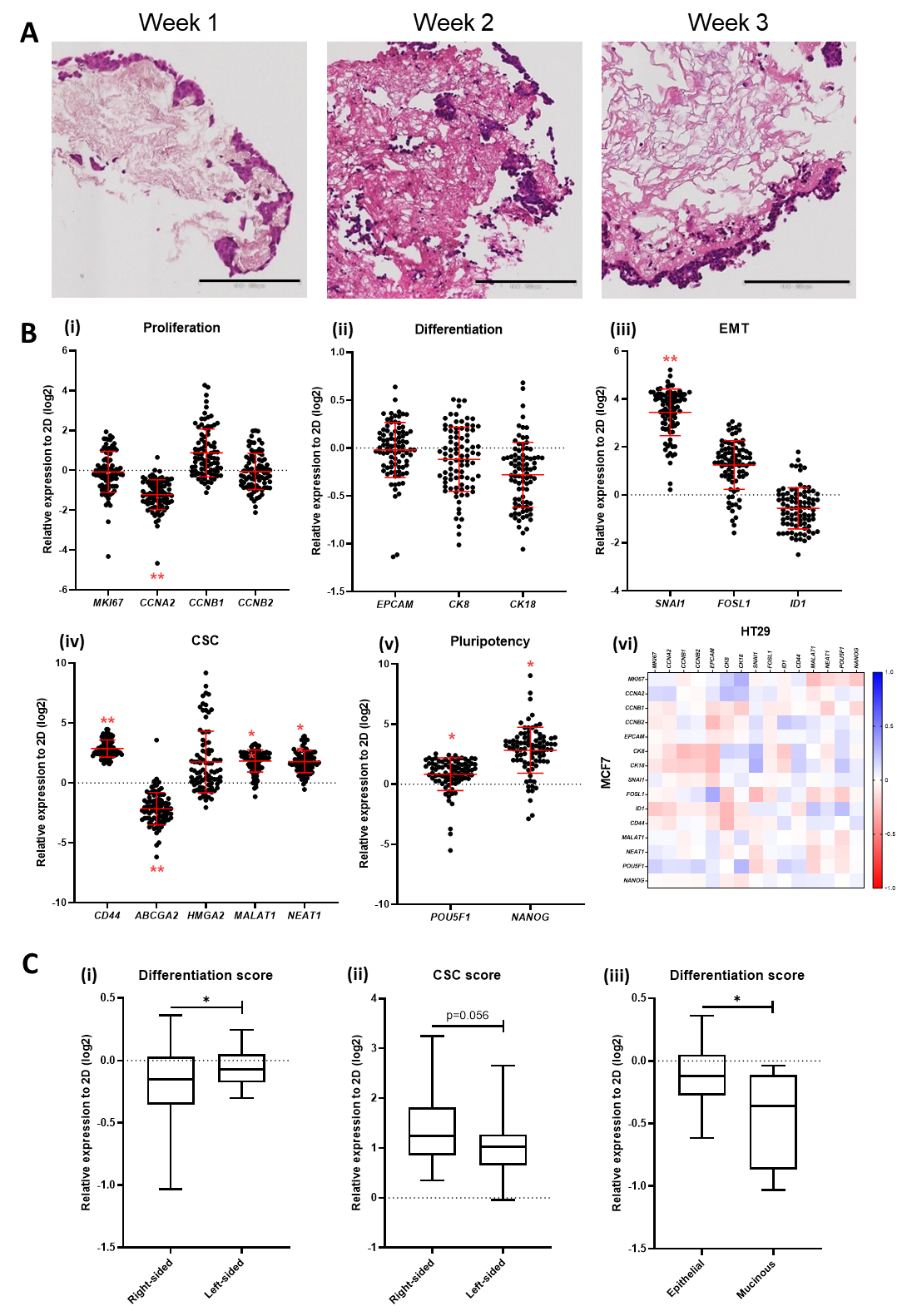


**Figure S3: Colorectal PDS repopulation with breast cancer cell line MCF7. (A)** Representative histological images of H&E stained 4.5 µm sections of colorectal PDS after 1-3 weeks of cell culture with MCF7 cells. Scale bars indicate 200 µm. **(B)** (i-v) Gene expression fingerprint of MCF7 cells after 3 weeks growth on colorectal PDS. Data is expressed relative to 2D expression levels. Red bars indicate mean ± SD (n=89). Dots indicate individual patients. *p<0.05; **p<0.01 (2D vs PDS, Mann-Whithney U test). (vi) Spearman’s rank correlation between colorectal PDS-induced gene expression in HT29 and MCF7 cells. **(C)** Associations of tumor clinical parameters with PDS-induced response of gene family scores in MCF7 cells. *p<0.05 (Mann-Whitney U test).

**
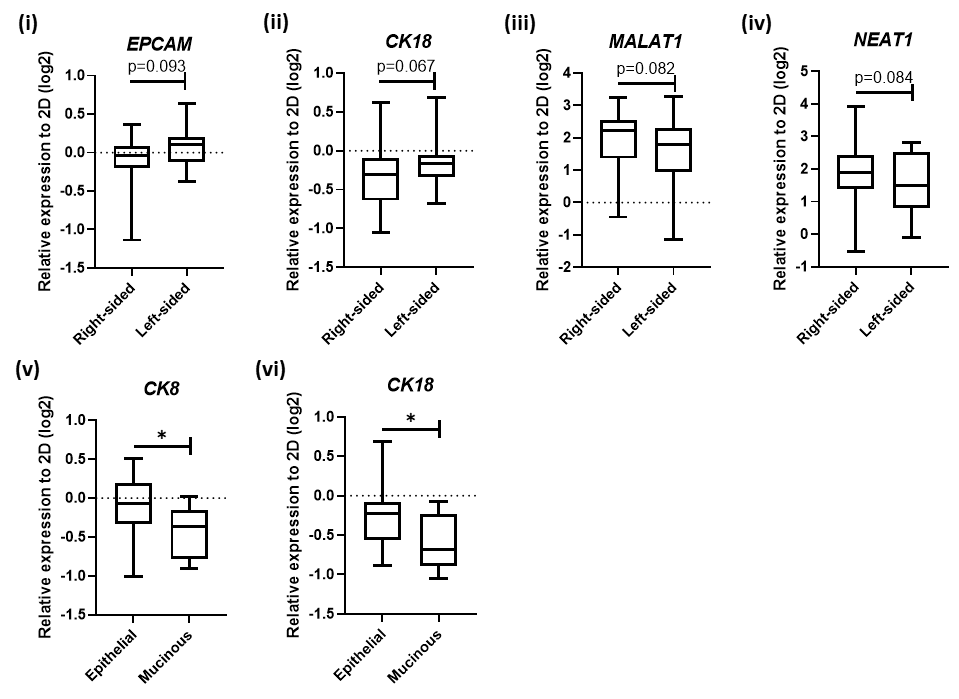
**

**Figure S4: Non-parametric statistical analysis of associations of PDS-induced MCF7 gene expression response with clinical parameter.** Associations of individual gene responses in PDS-grown MCF7 cells with (i-iv) tumor location and (v-vi) mucinous identity. *p<0.05; Mann-Withney U test.

# **SUPPLEMENTARY TABLES**

| ***Gender*** |  |  | ***Differentiation*** |  |
| --- | --- | --- | --- | --- |
| Male | 47 |  | Low | 18 |
| Female | 42 |  | Medium | 63 |
| ***Age*** |  |  | High | 1 |
| Min | 32 |  | Mucinous | 6 |
| Max | 93 |  | ***T-stage*** |  |
| Median | 73 |  | T1 | 14 |
| ***Tumor Location*** |  |  | T2 | 30 |
| Right-sided colon | 42 |  | T3 | 38 |
| Left-sided colon | 30 |  | T4 | 7 |
| Rectum | 17 |  | ***N-stage*** |  |
| ***Metastasis*** |  |  | N0 | 48 |
| Metastatic | 41 |  | N1 | 5 |
| Non metastatic | 48 |  | N2 | 36 |
| ***Recurrence*** |  |  |  |  |
| Recurrent | 12 |  |  |  |
| Non recurrent | 71 |  |  |  |

**Table S1: Patient demographic and clinical characteristics**

|  | ***CCNA2*** | ***MKI67*** | ***FOSL1*** | ***CCNB1*** | ***CK18*** | ***ID1*** | ***CCNB2*** | ***EPCAM*** | ***SNAIL*** | ***CD44*** | ***NANOG*** | ***POU5F1*** | ***MALAT1*** | ***NEAT1*** |
| --- | --- | --- | --- | --- | --- | --- | --- | --- | --- | --- | --- | --- | --- | --- |
| ***2D vs PDS*** | <0,0001 | <0,0001 | <0,0001 | <0,0001 | 0,0051 | 0,0045 | <0,0001 | 0,8557 | 0,9215 | 0,9857 | <0,0001 | <0,0001 | <0,0001 | <0,0001 |
| ***2D vs Matrigel*** | 0,0042 | <0,0001 | <0,0001 | <0,0001 | <0,0001 | 0,9917 | 0,7626 | 0,3662 | 0,0309 | 0,0975 | <0,0001 | <0,0001 | <0,0001 | <0,0001 |
| ***PDS vs Matrigel*** | 0,1872 | 0,9962 | 0,1221 | <0,0001 | 0,3029 | 0,0105 | 0,0004 | 0,6737 | 0,0109 | 0,0685 | 0,0171 | 0,4849 | 0,9863 | 0,3257 |

**Table S2: Statistical analysis of gene expression differences between 2D and 3D cultures.** The table reports p-values resulting from two-way ANOVA with Tukey’s post hoc test.

|  |  | ***Gender*** | |  | ***Tumor Location*** | |  | ***Differentiation*** | |  | ***T-stage*** | |  | ***Mucinous*** | |  | ***Metastasis*** | |  | ***N-stage*** | |  | ***Death by cancer*** | |
| --- | --- | --- | --- | --- | --- | --- | --- | --- | --- | --- | --- | --- | --- | --- | --- | --- | --- | --- | --- | --- | --- | --- | --- | --- |
| *Gene* |  | *p* | *p*  *(BH)* |  | *p* | *p*  *(BH)* |  | *p* | *p*  *(BH)* |  | *p* | *p*  *(BH)* |  | *p* | *p*  *(BH)* |  | *p* | *p*  *(BH)* |  | *p* | *p*  *(BH)* |  | *p* | *p*  *(BH)* |
| ***CCNA2*** |  | 0.340 | 0.037 |  | 0.951 | 0.095 |  | 0.043 | 0.011 |  | 0.048 | 0.005 |  | 0.539 | 0.079 |  | 0.923 | 0.089 |  | 0.020 | 0.005 |  | 0.504 | 0.053 |
| ***CCNB1*** |  | 0.459 | 0.063 |  | 0.948 | 0.089 |  | 0.160 | 0.016 |  | 0.476 | 0.063 |  | 0.128 | 0.021 |  | 0.955 | 0.100 |  | 0.040 | 0.011 |  | 0.592 | 0.063 |
| ***CCNB2*** |  | 0.727 | 0.089 |  | 0.782 | 0.058 |  | 0.184 | 0.021 |  | 0.254 | 0.026 |  | 0.047 | 0.016 |  | 0.781 | 0.079 |  | 0.091 | 0.016 |  | 0.711 | 0.079 |
| ***CD133*** |  | 0.951 | 0.100 |  | 0.250 | 0.042 |  | 0.350 | 0.037 |  | 0.569 | 0.074 |  | 0.523 | 0.074 |  | 0.263 | 0.032 |  | 0.903 | 0.095 |  | 0.945 | 0.100 |
| ***CD44*** |  | 0.054 | 0.011 |  | 0.482 | 0.047 |  | 0.028 | 0.005 |  | 0.185 | 0.021 |  | 0.676 | 0.089 |  | 0.525 | 0.058 |  | 0.594 | 0.058 |  | 0.837 | 0.095 |
| ***CK18*** |  | 0.454 | 0.058 |  | 0.489 | 0.053 |  | 0.449 | 0.053 |  | 0.075 | 0.016 |  | 0.513 | 0.068 |  | 0.040 | 0.005 |  | 0.533 | 0.053 |  | 0.022 | 0.011 |
| ***CK8*** |  | 0.395 | 0.053 |  | 0.920 | 0.084 |  | 0.266 | 0.032 |  | 0.426 | 0.047 |  | 0.310 | 0.047 |  | 0.171 | 0.021 |  | 0.761 | 0.079 |  | 0.189 | 0.037 |
| ***EPCAM*** |  | 0.869 | 0.095 |  | 0.220 | 0.032 |  | 0.518 | 0.063 |  | 0.435 | 0.053 |  | 0.177 | 0.037 |  | 0.539 | 0.068 |  | 0.136 | 0.021 |  | 0.508 | 0.058 |
| ***ETV1*** |  | 0.571 | 0.079 |  | 0.970 | 0.100 |  | 0.417 | 0.047 |  | 0.755 | 0.089 |  | 0.026 | 0.011 |  | 0.447 | 0.053 |  | 0.927 | 0.100 |  | 0.833 | 0.089 |
| ***FOSL1*** |  | 0.260 | 0.032 |  | 0.850 | 0.063 |  | 0.471 | 0.058 |  | 0.564 | 0.068 |  | 0.269 | 0.042 |  | 0.413 | 0.042 |  | 0.891 | 0.089 |  | 0.096 | 0.016 |
| ***FOXA2*** |  | 0.393 | 0.047 |  | 0.863 | 0.068 |  | 0.598 | 0.079 |  | 0.671 | 0.079 |  | 0.003 | *0.005 |  | 0.447 | 0.047 |  | 0.212 | 0.032 |  | 0.278 | 0.047 |
| ***ID1*** |  | 0.628 | 0.084 |  | 0.885 | 0.074 |  | 0.799 | 0.095 |  | 0.679 | 0.084 |  | 0.432 | 0.053 |  | 0.816 | 0.084 |  | 0.223 | 0.037 |  | 0.152 | 0.032 |
| ***MALAT1*** |  | 0.136 | 0.021 |  | 0.005 | *0.011 |  | 0.590 | 0.074 |  | 0.449 | 0.058 |  | 0.806 | 0.100 |  | 0.094 | 0.016 |  | 0.843 | 0.084 |  | 0.104 | 0.021 |
| ***MKI67*** |  | 0.470 | 0.068 |  | 0.888 | 0.079 |  | 0.251 | 0.026 |  | 0.288 | 0.032 |  | 0.172 | 0.032 |  | 0.528 | 0.063 |  | 0.394 | 0.042 |  | 0.802 | 0.084 |
| ***NANOG*** |  | 0.067 | 0.016 |  | 0.023 | 0.016 |  | 0.708 | 0.089 |  | 0.375 | 0.042 |  | 0.572 | 0.084 |  | 0.759 | 0.074 |  | 0.648 | 0.068 |  | 0.698 | 0.074 |
| ***NEAT1*** |  | 0.178 | 0.026 |  | 0.041 | 0.021 |  | 0.578 | 0.068 |  | 0.914 | 0.095 |  | 0.695 | 0.095 |  | 0.293 | 0.037 |  | 0.681 | 0.074 |  | 0.242 | 0.042 |
| ***NESTIN*** |  | 0.364 | 0.042 |  | 0.093 | 0.026 |  | 0.672 | 0.084 |  | 0.943 | 0.100 |  | 0.482 | 0.063 |  | 0.932 | 0.095 |  | 0.637 | 0.063 |  | 0.690 | 0.068 |
| ***POU5F1*** |  | 0.014 | 0.005 |  | 0.002 | *0.005 |  | 0.987 | 0.100 |  | 0.308 | 0.037 |  | 0.472 | 0.058 |  | 0.199 | 0.026 |  | 0.447 | 0.047 |  | 0.137 | 0.026 |
| ***SNAI1*** |  | 0.551 | 0.074 |  | 0.221 | 0.037 |  | 0.376 | 0.042 |  | 0.062 | 0.011 |  | 0.167 | 0.026 |  | 0.093 | 0.011 |  | 0.176 | 0.026 |  | 0.012 | 0.005 |

**Table S3: Non parametric statistical analysis of associations of tumor clinical parameters with PDS-induced response of cancer related genes in HT29 cells.** The table reports p-values (p) resulting from Mann-Whitney U test (Gender, Differentiation, Mucinous, Metastasis and Death by cancer) or Kruskal Wallis test (Tumor Location, T-stage and N-stage) and corresponding multitesting-corrected p-values (p(BH)). Multitesting correction was performed using the Benjamini-Hochberg method accepting 10% false discovery rate. Significant values after multitesting correction are indicated with *.

|  |  | ***Cancer Specific Survival*** | | |
| --- | --- | --- | --- | --- |
| *Covariate* |  | *Hazard Ratio* | *Confidence interval* | *P value* |
| ***T-stage*** |  | 2.73 | 1.53 – 4.87 | 0.001 |
| ***Tumor Location*** |  | 0.64 | 0.26 – 1.6 | 0.340 |
| ***Differentiation*** |  | 0 | 0 – 1.69x10^110^ | 0.905 |
| ***N-stage*** |  | 5357.06 | 0 – 2.59x10^62^ | 0.901 |
| ***PDS-induced EMT*** |  | 0.29 | 0.08 – 1.08 | 0.065 |

**Table S4: Multivariate Cox proportional hazard regression model predicting cancer specific survival.**

|  | ***Tumor***  ***Location*** | ***Gender*** | ***Different.*** | ***T-stage*** | ***Mucinous*** | ***Metastasis*** | ***N-stage*** | ***Death by cancer*** |
| --- | --- | --- | --- | --- | --- | --- | --- | --- |
| ***Proliferation*** | 0.834 | 0.536 | 0.721 | 0.683 | 0.095 | 0.421 | 0.723 | 0.116 |
| ***Differentiation*** | 0.048 | 0.457 | 0.963 | 0.935 | 0.022 | 0.651 | 0.448 | 0.548 |
| ***EMT*** | 0.537 | 0.616 | 0.557 | 0.129 | 0.631 | 0.231 | 0.437 | 0.661 |
| ***CSC*** | 0.056 | 0.285 | 0.881 | 0.167 | 0.112 | 0.065 | 0.066 | 0.862 |
| ***Pluripotency*** | 0.954 | 0.947 | 0.128 | 0.458 | 0.380 | 0.426 | 0.276 | 0.446 |

**Table S5: Non parametric statistical analysis of associations of tumor clinical parameters with PDS-induced response of cancer-related gene families in MCF7 cells.** The table reports p-values resulting from Mann-Whitney U test (tumor-location, gender, differentiation, mucinous, metastasis and death by cancer) or Kruskal Wallis test (T-stage and N-stage). Proliferation family included MKI67, CCNA2, CCNB1, CCNB2. Differentiation family included EPCAM, CK8, CK18. EMT family included SNAI1, FOSL1, ID1. CSC family included CD44, ABCGA2, HGMA2, MALAT1, NEAT1. Pluripotency family included POU5F1, NANOG.

| ***Antibody*** | ***Product code*** | ***Use and dilution*** |
| --- | --- | --- |
| ***β-actin*** | SC-1616 | Western Blot, 1:1000 |
| ***CCNA2*** | AB181591 | Western Blot, 1:20000 |
| ***CD44*** | AB16728 | Western Blot, 1:250 |
| ***Collagen IV*** | AB6586 | Immunohistochemistry, 1:500 |
| ***Fibronectin*** | AB 2413 | Immunohistochemistry, 1:500 |
| ***MKI67*** | M724029-2 | Immunohisyochemistry, 1:1000 |
| ***NANOG*** | NOVUS 104320 (5A10) | Western Blot, 1:1000 |
| ***POU5F1*** | H00005460-M05 | Western Blot, 1:1000 |
| ***SNAI1*** | CST C15D3 | Western Blot, 1:1000 |

**Table S6: List of primary antibodies and dilutions for immunohistochemistry and Western Blot use.**

| ***Gene*** | ***Forward sequence (5’-3’)*** | ***Reverse sequence (3’-5’)*** | ***Accession Number*** |
| --- | --- | --- | --- |
| ***ABCGA2*** | GGTGGAGGCAAATCTTCGTTA | GAGTGCCCATCACAACATCA | NM_004827.2 |
| ***CCNA2*** | AAGACGAGACGGGTTGC | GGCTGTTTACTGTTTGCTTTCC | NM_001237.4 |
| ***CCNB1*** | TTCTGGATAATGGTGAATGGAC | ATGTGGCATACTTGTTCTTGAC | NM_031966.3 |
| ***CCNB2*** | CGACCCTTGCCACTACACTT | TGACTTCCAATACTTCATTCTCTG | NM_004701.3 |
| ***CD133*** | GCCTCTGGTGGGGTATTTCT | TTTCCTTCTGTCGCTGGTG | NM_006017.2 |
| ***CD44*** | GAAGAAGGTGTGGGCAGAAGA | ACCATTTCCTGAGACTTGCTG | NM_000610.3 |
| ***CK18*** | CGAGAGACTGGAGCCATTACT | CGAGTCGTGTGATATTGGTGT | NM_000224.2 |
| ***CK8*** | CGACAAGGTAGAGCTGGAGTCT | CGAGCACCACAGATGTGTCCGA | NM_001256282.1 |
| ***EPCAM*** | CAGGAAGAATGTGTCTGTGAAAACT | TTCATTTCTGCCTTCATCACC | NM_002354.2 |
| ***ETV1*** | ACCTGTGTTGTCCCAGAAAAAT | TCCTCGCCGTTGGTATGTG | NM_004956.4 |
| ***FOSL1*** | GCAGGCGGAGACTGACAA | GGGGAAAGGGAGATACAAGG | NM_001300855.2 |
| ***FOXA2*** | AGGAGGAAAACGGGAAAGAA | CAACAACAGCAATGGAGGAG | NM_021784.4 |
| ***HMGA2*** | CCGTCCACTTCAGCCCAG | GGACTCTTGTTTTTGCTGCCT | NM_003483.4 |
| ***ID1*** | CTGAGGGAGAACAAGACCGAT | CCCCCTAAAGTCTCTGGTGA | NM_002165.4 |
| ***MALAT1*** | CGACGAGTTGTGCTGCTATC | TCCTCCAAACCCCAAGACCA | NR_002819.4 |
| ***MKI67*** | TGGGTCTGTTATTGATGAGCC | CATCAGGGTCAGAAGAGAAGC | NM_004360.4 |
| ***NANOG*** | CCTATGCCTGTGATTTGTGG | AAGTGGGTTGTTTGCCTTTG | NM_024865 |
| ***NEAT1*** | GCCTTCTTGTGCGTTTCTCG | CCCTCCCAGCGTTTAGC | NR_028272.1 |
| ***NESTIN*** | TTGGAACAGAGGTTGGAG | GCTGAGGGACATCTTGAG | NM_006617.1 |
| ***POU5F1*** | CGAAAGAGAAAGCGAACCAG | AACCACACTCGGACCACATC | NM_002701 |
| ***SNAI1*** | TAATCCAGAGTTTACCTTCCAGCA | AGCCTTTCCCACTGTCCTCA | NM_005985.3 |

**Table S7: List of primer sequences for qPCR**
